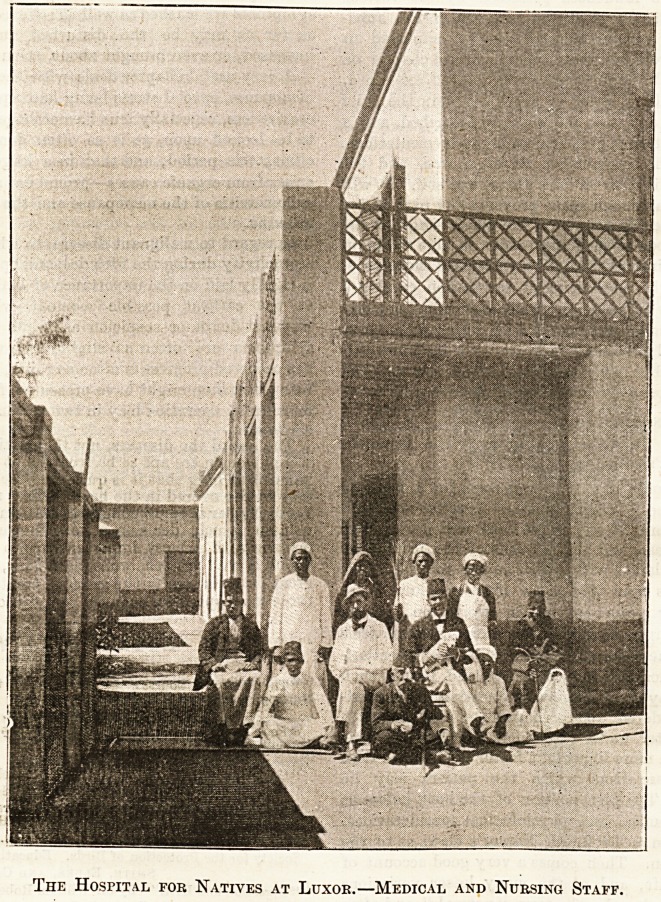# Hospital Construction

**Published:** 1897-05-01

**Authors:** Conrad W. Thies

**Affiliations:** Secretary Royal Free Hospital


### The Institutional Workshop.

### HOSPITAL CONSTRUCTION.

### THE HOSPITAL FOR NATIVES AT LUXOR, UPPER EGYPT.

By Conrad W. Thies, Secretary Royal Free Hospital.

This hospital owes its establishment to the generosity of Mr. John M. Cook (head of the well-known firm of Messrs. Thomas Cook and Son), Sir John Brunner, and other travellers in Egypt. It was formally opened by the late Khedive, Tewfik Pasha, in 1891. Prior to this date there was no institution for affording medical treatment to the dwellers in the thickly-populated plains of Thebes and Upper Egypt, who, in cases of serious illness or accident, had to seek medical relief in the far-distant towns of Lower Egypt.

The hospital is pleasantly situated on the outskirts of the village of Luxor, on land raised well above the highest level of the annual inundations of the Nile. The buildings are entirely surrounded by a high wall, which shelters them from the sands of the neighbouring desert. They consist of an administration block, four detached wards, kitchens, lavatories, &c., each under a separate roof. In accordance with the custom of Mohammedan countries, a ward is walled off from the rest of the buildings, and is set apart as a hareem for female patients.

In the main block are waiting-rooms for patients, dispensary, and operating-room, above which are the quarters of the resident medical officer.

The rest of the buildings are built on the pavilion principle, the foundations and floors being of concrete and cemented bricks, to keep out the moisture which arises from the soil during the Nile inundations. Careful attention has been given to the sanitary arrangements, which are always difficult to maintain in an efficient condition in a country like Egypt, and when dealing with natives who are practically devoid of any ideas as to the necessity for such precautions.

The hospital provides 26 beds, and over 300 patients were admitted last year, while the out-patients average about 7,000 per annum.

The Hospital for Natives at Luxor.—Medical and Nursing Staff.

The admission is practically free to all poor natives; those in better circumstances may, however, be received at the discretion of the medical officer upon payment of a sufficient sum to cover the cost of their maintenance.

In making the preliminary arrangements for building and starting the hospital, the founders were fortunate in having the advice of Dr. Milton, medical superintendent of the great hospital of Kasr el Aini at Cairo, who has had a large experience of hospital requirements in Egypt. The management is entrusted to Dr. Longmore, who spends the winter months of each year in Luxor. He is ably assisted by Dr. Mahomet el Hakim, the resident medical officer, who lives on the hospital premises throughout the year, and is responsible for the work generally.

The staff consists of three native male attendants, and one female attendant. During the winter season an English nurse resides at the Luxor Hotel, and renders assistance and advice when required.

In going over the hospital with Dr. Mahomet, I was impressed with the evidence of the care and thoroughness with which every detail of the work was carried out, and by the large proportion of serious and difficult cases which were under treatment. Several of the medical patients were suffering from a disease—anchylostomiasis—caused by the presence of minute intestinal parasitic worms, which arises from the habits and mode of life of the fellaheen, and has been since the earliest times one of the veritable plagues of Egypt. About one-eighth of the medical patients are admitted suffering from pulmonary disorders, but it is a fact that pleurisy, pneumonia, and bronchitis are not uncommon amongst the thinly clad natives of Upper Egypt, and more especially amongst the negroid tribes of the Soudan during the winter months.

One-fourth of the in-patients are suffering from diseases of the eye, which are exceedingly prevalent throughout the country owing to the ever-present plagues of flies and dust combined with the absence of personal cleanliness. It is no uncommon sight in the Nile villages to see young children with their dusty faces simply covered with flies, of which neither they or their parents appear to take the slightest notice.

Amongst the surgical patients a large proportion are accident cases. The large number of wounds and fractures arise from the primitive and comparatively savage conditions of life in the villages; very severe wounds frequently result from the knives or heavy sticks so readily used by the natives in their quarrels. Many cases of vesical calculus occur, an interesting feature being the large number of children under ten years of age suffering from this disease.

In conclusion, I may say I was most favourably impressed by what I saw at this excellent hospital, which must be of immense service to the poor dwellers on the banks of the Nile, and ought to recommend itself as deserving support from all Egyptian travellers.

## Figures and Tables

**Figure f1:**